# Relationship of Body Composition with the Strength and Functional Capacity of People over 70 Years

**DOI:** 10.3390/ijerph17217767

**Published:** 2020-10-23

**Authors:** Fredy Alonso Patiño-Villada, Jerónimo J González-Bernal, Josefa González-Santos, José Antonio de Paz, Maha Jahouh, Juan Mielgo-Ayuso, Ena Monserrat Romero-Pérez, Raúl Soto-Cámara

**Affiliations:** 1 University Institute of Physical Education and Sports, University of Antioquia, 76270 Antioquia, Colombia; fredpa18@hotmail.com; 2 Department of Health Sciences, University of Burgos, 09001 Burgos, Spain; mjx0002@alu.ubu.es (M.J.); rscamara@ubu.es (R.S.-C.); 3 Institute of Biomedicine, University of León, 24071 León, Spain; japazf@unileon.es; 4 Department of Biochemistry, Molecular Biology and Physiology, Faculty of Health Sciences, University of Valladolid, 42004 Soria, Spain; juanfrancisco.mielgo@uva.es; 5 Department of Sports and Physical Activity Sciences, University of Sonora, 83067 Sonora, Mexico; ena.romero@unison.mx

**Keywords:** muscle power, functional fitness, muscle mass, fat mass, bone mass, elderly, Spain

## Abstract

Background: Aging is a multifactorial physiological phenomenon, in which a series of changes in the body composition occur, such as a decrease in muscle mass and bone mineral density and an increase in fat mass. This study aimed to determine the relationship of muscle mass, osteoporosis, and obesity with the strength and functional capacity of non-dependent people over 70 years of age. Methods: A cross-sectional study was designed, whose study population was all people aged over 70 years, living independently and attending academic and recreational programs. Muscle strength and functional capacity of the participants were assessed by isometric exercises of lower and upper limbs and by four tests taken from the Senior Fitness Test, respectively. Bone mineral density, total mass, fat mass, total lean mass, arms lean mass, legs lean mass, and appendicular lean mass (ALM) was calculated by dual energy X-ray absorptiometry. Differences in muscle strength and functional capacity, according to the sex, muscle mass, mineral bone density and fat mass, were measured by χ^2^ test, independent samples Student’s t-test, analysis of covariance and a 2-factor analysis of covariance; Results: 143 subjects were included in the study group. Men and women with an adequate amount of ALM adjusted for body mass index (BMI) had a maximal dynamic biceps strength in a single repetition, a maximal isometric leg extension strength, a maximal dynamic leg extension strength in a single repetition, a maximum right hand grip strength and maximum hand grip strength (the highest). Significantly higher values were observed in the maximal isometric biceps’ strength in men with osteoporosis. Obese men had less isometric strength in the biceps and took longer to perform the chair stand test; Conclusions: Men and women with an adequate amount of ALM adjusted for BMI obtained better results in tests of muscle strength and functional capacity. However, osteoporosis and obesity are not related to these parameters.

## 1. Introduction

Aging is a complex multifactorial biological phenomenon. It generates changes in body composition [1], including a decrease in muscle and bone mass and bone mineral density and an increase in fat mass [2], which produce a variety of physiological consequences on health [3]. Further, early in, these changes in body composition are associated with a loss of muscle strength, which can affect the functional status of the elderly and their quality of life, causing the loss of their independence [4] and, therefore, they may end up going to residential centers [5]. In this sense, muscle strength is excellent parameter for predicting independence and mobility in the elderly [6,7]. Decreased muscle strength are associated with overall strength, gait, and balance problems that increase the risk of falls [8,9]. Therefore, the measurement of muscle strength can be used to visualize the ability of the elderly to live independently. Likewise, functional status and disability are components that are related to the progression of the aging process [10].

Low levels of muscle strength, activity, or functionality has been associated with reduced levels of muscle mass and an increased risk of morbidity and mortality [11]. Loss of muscle mass can affect functional capacity, due to a positive association between muscle mass and function of the lower limb [2,6] and negative with, the difficulties in activities of daily living, the risk of using a walking stick or a walker and the history of previous falls [12].

Although a correlation between muscle strength and bone mineral density has been observed in women with early menopause [13], there is conflicting evidence on the relationship between decreased muscle strength indicated by isometric and isokinetic tests and decreasing levels of bone mineral density [14,15,16]. In this line, some studies have reported little or no association between these factors [2]. However, other studies have shown a strong concomitant decline in muscle strength and bone mineral density during old age, suggesting that these levels are closely and progressively related to the physiology of advanced age [7]. The loss of bone mass is a powerful risk factor for fragility fractures that, in turn, cause loss of functional capacity, dependence and an increased risk of institutionalization [7]. Therefore, determining the relationship between isometric and isokinetic muscle strength and the decline in bone mineral density at an individual’s age is critical for the prevention of osteoporosis, suggesting that early initiation of preventive care should start at the beginning of muscle deterioration, even before a significant loss of bone mineral density [17,18,19].

On the other hand, obesity is associated with functional limitations in muscle performance and with a greater probability of developing a functional disability in mobility, strength, posture or dynamic balance [20]. Although most studies agree that absolute strength is higher in obese compared to non-obese adults, strength is lower when it is normalized to weight [21]. This may be due to a higher state of systemic inflammation [22] and to the inability to regenerate skeletal muscle in the obese individuals [23]. However, the association between strength and obesity is not very clear in old people.

As in most developed countries, in Spain the number of people over 70 years has increased considerably lately [24]. Considering that changes in body composition during aging are related to muscle strength and negatively influence the functionality and well-being of older people [25,26], accurate information is required to link both variables in order to propose different preventive programs. The objective of these programs will be to ensure the best possible body composition in older people that allows them to live independently without the need for institutionalization. Therefore, this study aimed to determine the relationship of muscle mass, osteoporosis and obesity with the strength and functional capacity of non-dependent people over 70 years of age.

## 2. Materials and Methods 

### 2.1. Study Design-Participants

A cross-sectional study was designed, whose study population was all people aged over 70 years, residing in Leon (Spain) and living independently, who attended academic and recreational programs from three community centers in Leon. Exclusion criteria were: Cognitive impairment, heart failure (grades II–IV), ischemic heart disease, and uncontrolled musculoskeletal problems that would prevent the completion of the tests.

### 2.2. Procedure-Data Collection

Academic and recreational programs, developed in three community centers, were used to recruit the participants. In academic programs, the participants were enrolled in activities not regulated in the university process, specifically aimed at older people; while in recreational programs, leisure and free time activities, not related to exercise or sport, were carried out. All possible participants of the study, who met the inclusion criteria, were referred by the center physicians. Participants were selected by a non-probabilistic convenience sampling. Prior to starting the study, informative meetings were held, in which the objective and methodology of the study as well as its voluntary nature were explained to them. They were invited to participate in the study, having to give their written informed consent in case of acceptance.

Before starting the assessments, participants filled out the Physical Activity Readiness Questionnaire [27]. If any of the answers were “yes”, a doctor was consulted to make sure there were no problems to do the exercises. The participants visited the laboratory on three occasions. On the first occasion, the clinical history and densitometry were taken, followed by a familiarization session (submaximum) with the instruments and procedures for strength evaluation. The strength evaluation was carried out by the same evaluators during the second and third visit, with an interval of 3 to 5 days, following a previously established chronological order. The approximate duration of each session was 45–50 min per subject. These sessions were held in facilities with good lighting, ventilation and free from distractions, where facilities where the necessary measuring instruments and machines were available.

Ethical approval was obtained from the Bioethics Committee of the University of León (Spain) (Reference ULE-032–2015) and the study respected the principles of the Declaration of Helsinki. 

### 2.3. Main Outcomes-Instrument

The muscle strength and the functional capacity of the participants were the main outcomes from the study. Functional performance was assessed according to four tests taken from the Senior Fitness Test—arm curl, chair stand, step in place, and 8 foot up and go—which have been validated for the evaluation of functional fitness in older adults [28,29,30,31]. Isometric hand grip strength of both the dominant and the non-dominant side were performed with each subject sitting, the shoulder at 90° and the elbow in full extension using a Jamar dynamometer (Promedics, Blackburn, UK). Two trials for each hand were performed and the highest value of the strongest hand was used in the analyses. Maximum voluntary isometric strength of quadriceps was measured for both legs using a load cell (Globus Ergo System, software IsoMetric 20.40 Test, Codognè Italy) in a leg extension machine (BH Fitness Nevada Pro-T, Madrid, Spain). On command, the subject performed an isometric quadriceps extension (as fast as possible) at 90° of knee flexion during five seconds. Two trials were performed; the highest result of the quadriceps strength was used. 

Other data collected were age, gender, bone mineral density and body composition. The last two variables were assessed by dual energy X-ray absorptiometry (Lunar Prodigy-GE, Software Encore 2009^®^ version 12.1-, Diegem, Belgium). Total mass, fat mass, total lean mass, arms lean mass, legs lean mass, and appendicular lean mass (ALM) measured in kilograms (kg) were obtained for each participant. Height was measured in centimeters (cm) once by using a body meter (SECA Model 208), which has an accuracy of up to 0.05 cm. Low muscle mass was defined according to ALM adjusted for body mass index (BMI) as a threshold of 7.26 kg/m^2^ for men and 5.50 kg/m^2^ for women [12]. Obesity and osteoporosis was defined according to international standards [32,33] 

### 2.4. Statistical Analysis

Descriptive analyses were conducted to describe the sample. Categorical variables were expressed as absolute frequencies and percentages, while the continuous variables were expressed in terms of mean and standard deviation (SD). The compliance of the normality criteria of the quantitative variables was assessed by Kolmogorov-Smirnov test. Differences between men and women in muscle strength and functional capacity, according to the amount of ALM adjusted for BMI, the presence or not of osteoporosis and the body fat percentage, were measured by χ^2^ test. On the other hand, the association between anthropometric and body composition variables in men and women was assessed using independent samples Student’s t-test. The mean levels of physical tests of men and women were compared according to the amount of ALM adjusted for BMI, the presence or not of osteoporosis and the body fat percentage, using an analysis of covariance. In addition, with the aim of knowing if sex was a determining factor in each of the categories, a 2-factor analysis of covariance (study categories x sex) was performed, adjusting all analyzes by age. Effect sizes were calculated using partial eta squared (η^2^ *p*) and interpreted according to the following criteria: If 0 ≤ η^2^ *p* < 0.05, there is no effect; if 0.05 ≤ η^2^ *p* < 0.26, the effect is minimal; if 0.26 ≤ η^2^ *p* < 0.64, the effect is moderate; and if η^2^ *p* ≥ 0.64, the effect is strong. For the analysis of statistical significance, a value of *p* < 0.05 was established. Statistical analysis was performed with SPSS version 25 software (IBM-Inc, Chicago, IL, USA).

## 3. Results

The number of subject, who met the eligibility criteria and voluntarily agreed to participate in the study, was 143. The sample consisted of 94 women, whose age, weight and height mean was 75.14 years (SD ± 3.85), 63.43 kg (SD ± 9.36), and 152.21 cm (SD ± 5.74), respectively. The age, weight, and height mean of the 49 men included was 74.84 years (SD ± 3.84), 76.42 kg (SD ± 10.88) and 165.84 cm (SD ± 6.86) respectively.

The data on body composition, distribution of weakness (muscle mass), osteoporosis and obesity of the sample, according to sex, are shown in Table 1. Men presented values of total lean mass (kg), ALM (kg), ALM adjusted for BMI, bone mass (kg), femoral neck and lumbar spine bone mineral density (g/cm^2^), femur neck, and lumbar spine T-score higher than women, being this differences statistically significant (*p* < 0.05). However, women obtained statistically higher values (*p* < 0.05) than men in fat mass (kg), fat mass (%), and femur neck and lumbar spine Z-score. In the total sample there was a statistically greater distribution (*p* < 0.05) of obese, people with adequate lean mass based on ALM adjusted for BMI and people without osteoporosis.

When analyzing the relationship of muscle strength and functional capacity with the amount of the ALM adjusted for BMI, it was observed that both men and women with an adequate amount had a maximal dynamic biceps strength in a single repetition (kg), a maximal isometric leg extension strength (kg), a maximal dynamic leg extension strength in a single repetition (kg), a maximum right hand grip strength (kg), and maximum hand grip strength (the highest). For their part, women with an adequate amount also had a maximal isometric biceps strength (kg) (*p* < 0.001) and a maximum left hand grip force (kg) (*p* ≤ 0.001), as well as the best functional capacity measured by the arm curl test (repetition) (*p* < 0.001), the step-in-place (steps) (*p* = 0.043), and the 8 foot up and go test (s) (*p* = 0.003). The behavior of the arm curl test was different between categories and sexes (*p* = 0.018; η^2^ *p* = 0.040) (Table 2).

Having osteoporosis was not related to changes in muscle strength or functional capacity, according to sex. However, in men with osteoporosis, significantly higher values were observed in the maximal isometric biceps strength (kgs) in relation to those other men without osteoporosis (*p* = 0.013). Finally, it should be noted that the behavior of the maximal isometric biceps’ strength test was different between categories and sexes (*p* = 0.020; η^2^ *p* 0.039) (Table 3).

When comparing the muscle strength and functional capacity of men and women older than 70 years according to their fat percentage (normal or obesity), no statistically significant differences were observed between individuals. Obese men had less isometric strength in the biceps (kg) (*p* = 0.043) and took longer to perform the chair stand test (*p* = 0.039) in relation to normal subjects (Table 4).

## 4. Discussions

This study was proposed in order to identify the relationship of muscle mass, osteoporosis and obesity mass with the muscle strength and functional capacity of non-dependent people over 70 years. The main result showed that although osteoporosis and obesity did not show relation with the muscle strength and functional capacity, men and women with an adequate amount of ALM adjusted for BMI have obtained better results in tests of muscle strength and functional capacity. In turn, women have also presented a higher maximal isometric biceps strength (kg) and a higher maximum left hand grip force (kg) as well as the greater functional capacity measured by the arm curl test, the step-in-place, and the 8 foot up and go test (s). Low muscle mass in non-dependent people older than 70 years is an issue which has been poorly investigated in studies. This fact makes it difficult to establish comparisons between other variables and samples. Even so, it can be affirmed that in independent people over 70 years obesity and osteoporosis do not related to the people’s functional capacity, although their muscle mass does.

Although changes in body composition are the consequence of a physiological and multifactorial process, which occurs throughout the aging process, even in healthy people, lifestyle plays a particularly relevant role on muscle, bone and lean mass. Specifically, the World Health Organization (WHO) advocates nutrition and physical activity as factors that greatly influence the body composition of older people [34]. A large number of studies have shown that both physical activity, preferably through accelerometers, and specific training programs are capable of reversing, at least partially, changes in body composition in sedentary older people [35,36]. This seems to indicate that an active lifestyle preserves muscle, fat and bone mass at healthy levels [37,38]. In addition, physically active people have a lower risk of suffering pathologies associated with body composition than those with a sedentary lifestyle [39], which correlates with the results obtained in this study.

Like Krause et al. [40], who carried out an investigation with a sample of 33 people aged over 65 years, whose objective was to determine the relationship of the fat-free mass index with the anthropometric, gait, balance, and strength measures, no significant relationship between body composition and 8 foot up and go test results has been observed in this study. However, statistically significant differences have been obtained in elbow flexion between men and women. Based on these results, there is no relationship between muscle mass and functionality of the lower limbs, although there is it with the strength of the upper limbs. A possible explanation for these findings is that both isometric strength and muscle power decrease over the years due to a loss of muscle mass, the inability of the muscle to generate force in a normal way and a decrease in speed of muscle contraction.

On the other hand, people with osteoporosis have presented slightly higher scores in some muscle strength and functional capacity tests, which, with the exception of the isometric force of biceps in the men, did not statistically significant. This poor association between osteoporosis and muscle strength has also been reported in other studies conducted in older populations and would seem to contradict recommendations to train strength to decrease the prevalence of osteoporosis or improve bone mineral density [41]. Some studies find no difference in strength between groups with osteoporosis and those without, despite finding a correlation between strength and bone mineral density [42]. A possible explanation for these results is that bone mineral density depends on multiple factors such as dietary intake of calcium and vitamin D, calcium absorption and incorporation, or bone mechanical stress. [43]. In general population studies, there is a weak significant correlation between muscle strength and bone mineral density (Pearson’s r 0.15–0.45), varying according to age of the sample and the part of the body analyzed (hip, spine, other areas) [41,44]. A significant value of Pearson’s r does not imply a causal relationship between these two variables, despite the fact that in the same person the increase in strength tends to be associated with a certain increase in bone mineral density [45,46].

A study by Villa et al. [47] was the first to suggest the existence of a distinct and non-linear relationship between the reduction in muscle strength and the loss of bone mineral density by age. This relationship may be useful as a prognostic indicator of osteoporosis, as well as the conventional assessment of muscle mass and body weight [47]. It can also be used as an early indicator of the susceptibility of women to progressive loss of bone mineral density during early menopause if they exhibit noticeable muscle loss during this period. In men with osteoporosis, more biceps muscle strength has been observed (*p* = 0.013), although the sample estimate is very low (η^2^ *p* = 0.039). This result may be of particular importance in routine osteoporosis detection and prevention strategies, as early initiation of preventive care, perhaps they should start before the onset of muscle deterioration, even before significant loss of bone mineral density. These data are similar to those obtained in a multicenter study carried out with a sample of people older than 70, institutionalized, with preserved walking ability. The prevalence of osteoporosis was within the range considered normal in nursing homes (17.7–73.3%) [48]. 

The muscle strength and functional capacity of people aged older 70 years have not been related to their fat percentage. In an experimental study with elderly women aged 76 to 78 years, it has been observed that intense muscle strength training can induce skeletal muscle hypertrophy, reduce the relative amount of intramuscular fat, and improve voluntary isometric contraction of the knee extensor muscles [49].

However, despite this beneficial effect of weighted exercise, obese individuals have lower postural stability and lower isometric strength in the biceps, data consistent with those obtained in this study [50,51]. Thus, although comparisons can be made between the additional burden of a hypergravity environment versus the excess burden experienced by an obese individual, the detrimental consequences of obesity appear to outweigh any potential benefits of a higher burden. However, increased levels of physical activity can promote increased muscle strength, reducing the detrimental consequences of obesity [52].

The study findings must be considered within the context of their limitations. It is a cross-sectional study, which does not allow us to determine a causal relationship between the variables. The results obtained in this study require confirmation in a higher sample, for generalizability purposes. So, it would be necessary to carry out more controlled studies in the future, with a wider variation of participants in aspects such as age, ethnicity, culture, severity of osteoporosis or level of obesity. The sample selection by a non-randomized convenience sampling procedure may make the results are not representative to the rest of the population. Also, the existence of a reduce number of studies on this issue makes it difficult to contrast the results obtained. These limitations can reduce the representativeness of the findings and may have influenced the results of the study. 

## 5. Conclusions

Independent people over 70 have an adequate muscle mass. Osteoporosis and obesity do not relate to the muscle strength and functional capacity of people, unlike their muscle mass. In recent years, the presence of unbalanced levels of muscle, bone, and fat mass have become a public health problem, and could become a worldwide epidemic [53]. This situation has detrimental implications for the functioning of skeletal muscle, being unknown the specific adaptations by gender and age in the presence of adiposity and low ALM.

New research is needed to study the possible relationship between the percentage of body fat, agonist muscle activation (using the interpolated contraction technique) and antagonist coactivation (using surface electromyography) in elderly with low amount of the ALM adjusted for BMI, according to their weight, and analyze its influence on strength and functional capacity.

## Figures and Tables

**Table 1:** Descriptive characteristics of sample.

-	Total (n = 143)	Female (n = 94)	Male (n = 49)	*p*-Value
Body Mass Index (BMI) (kg/m^2^)	27.51 ± 3.57	27.41 ± 3.93	27.71 ± 2.79	0.633 ^#^
Total lean mass (kg)	42.14 ± 8.42	36.93 ± 3.48	52.13 ± 5.59	<0.001 ^#^
Appendicular lean mass (ALM) (kg)	17.64 ± 3.92	15.26 ± 1.72	22.23 ± 2.66	<0.001 ^#^
ALM adjusted for BMI (ALM/BMI)	0.65 ± 0.14	0.56 ± 0.08	0.81 ± 0.09	<0.001 ^#^
Fat mass (kg)	23.41 ± 7.20	24.45 ± 7.20	21.44 ± 6.86	0.017 ^#^
Fat mass (%)	34.31 ± 7093	37.83 ± 6.54	27.54 ± 5.65	<0.001 ^#^
Bone mass (kg)	2.29 ± 0.50	2.01 ± 0.32	2.82 ± 0.34	<0.001 ^#^
Femoral neck bone mineral density (g/cm^2^)	0.82 ± 0.11	0.79 ± 0.10	0.87 ± 0.11	<0.001 ^#^
Lumbar spine bone mineral density (g/cm^2^)	1.03 ± 0.16	0.98 ± 0.15	1.12 ± 0.15	<0.001 ^#^
Femur neck T-score	−1.56 ± 0.84	−1.58 ± 0.84	−1.53 ± 0.83	0.713 ^#^
Lumbar spine T-score	−1.39 ± 1.30	−1.68 ± 1.25	−0.83 ± 1.23	<0.001 ^#^
Femur neck Z-score	0.15 ± 0.79	0.23 ± 0.79	−0.01 ± 0.78	0.085 ^#^
Lumbar spine Z-score	0.24 ± 1.30	0.51 ± 1.21	−0.29 ± 1.31	<0.001 ^#^
Lean mass based on ALM adjusted for BMI (n (%))				
• Low	49 (34.3)	27 (28.7)	22 (44.9)	<0.001 ^&^
• Adequate	94 (65.7)	67 (71.3)	27 (55.1)	
Osteoporosis status (n (%))				
• No	102 (71.3)	62 (66.0)	40 (81.6)	<0.001 ^&^
• Yes	41 (28.7)	32 (34.0)	9 (18.4)	
Obesity according body fat percentage (n (%))				
• Obese	107 (74.8)	75 (79.8)	32 (65.3)	<0.001 ^&^
• Normal	36 (25.2)	19 (20.2)	17 (34.7)	

Data are mean ± standard deviation; ^#^ *p*-value from one-way analysis of covariance; ^&^ *p*-value from χ^2^ test; *p* < 0.05 indicates statistical significance.

**Table 2:** Strength and functional capacity between low or adequate amount of appendicular lean mass (ALM) adjusted for body mass index (BMI) in men and women older than 70 years.

Sex	ALM Adjusted for BMI		*p*-Value	η^2^ *p*	*p*-Value (Gxsex)	η^2^ *p*
	Low	Adequate				
**Maximal isometric biceps strength (kg)**						
Women	16.30 ± 4.25	21.26 ± 5.40	<0.001	0.156	0.508	0.003
Men	31.90 ± 7.34	35.45 ± 8.23	0.190	0.037		
**Maximal dynamic biceps strength—1 RM (kg)**						
Women	11.13 ± 3.55	14.98 ± 5.22	0.001	0.108	0.240	0.010
Men	30.45 ± 8.75	36.96 ± 8.45	0.018	0.116		
**Maximal isometric leg extension strength (kg)**						
Women	47.91 ± 14.83	61.67 ± 14.49	<0.001	0.147	0.755	0.001
Men	78.52 ± 16.97	94.45 ± 26.25	0.031	0.097		
**Maximal dynamic leg extension strength—1 RM (kg)**						
Women	45.61 ± 12.78	54.98 ± 12.32	0.002	0.096	0.805	0.000
Men	68.68 ± 15.02	79.41 ± 14.86	0.027	0.102		
**Maximum hand grip strength (left) (kg)**						
Women	18.76 ± 3.49	21.99 ± 3.86	<0.001	0.126	0.914	0.000
Men	32.14 ± 7.48	35.22 ± 6.78	0.235	0.030		
**Maximum hand grip strength (right) (kg)**						
Women	19.93 ± 3.79	23.06 ± 4.41	0.003	0.094	0.219	0.011
Men	33.23 ± 7.16	38.70 ± 6.32	0.012	0.130		
**Maximum hand grip strength (the highest) (kg)**						
Women	20.33 ± 3.52	23.72 ± 4.08	0.001	0.125	0.341	0.007
Men	33.95 ± 7.01	39.07 ± 6.09	0.016	0.120		
**Arm curl test (rep)**						
Women	15.85 ± 3.75	19.57 ± 3.54	<0.001	0.175	0.018	0.040
Men	16.23 ± 2.98	17.04 ± 2.78	0.448	0.013		
**Chair stand test (rep)**						
Women	17.11 ± 4.35	17.27 ± 2.73	0.940	0.000	0.262	0.009
Men	17.50 ± 4.67	16.33 ± 3.17	0.140	0.047		
**Step-in-place (steps)**						
Women	96.44 ± 24.59	105.04 ± 12.61	0.043	0.044	0.758	0.001
Men	105.18 ± 21.63	111.93 ± 18.00	0.301	0.023		
**8 foot up and go test (s)**						
Women	5.83 ± 1.52	5.11 ± 0.67	0.003	0.093	0.094	0.020
Men	5.24 ± 1.10	5.14 ± 1.30	0.831	0.001		

Data are presented as mean ± standard deviation; All analyzes are adjusted by age; *p*-value: differences between groups (low vs normal) in each sex by one-way ANOVA; *p*-value (Gxsex): group-by-sex interaction (*p* < 0.05, all such occurrences); Two-factor repeated-measures ANOVA; 1 RM: one maximal repetition; rep: repetition; s: seconds.

**Table 3:** Strength and functional capacity between no or yes osteoporosis in men and women older than 70 years.

Sex	Osteoporosis		*p*-Value	η^2^ *p*	*p*-Value (Gxsex)	η^2^ *p*
	No	Yes				
**Maximal isometric biceps strength (kg)**						
Women	19.71 ± 5.42	20.08 ± 5.88	0.670	0.002	0.020	0.039
Men	32.65 ± 7.29	39.23 ± 9.01	0.013	0.126		
**Maximal dynamic biceps strength—1 RM (kg)**						
Women	14.06 ± 5.15	13.51 ± 5.04	0.727	0.001	0.270	0.009
Men	33.60 ± 5.99	36.00 ± 9.90	0.307	0.23		
**Maximal isometric leg extension strength (kg)**						
Women	58.65 ± 16.91	55.91 ± 13.47	0.505	0.005	0.627	0.002
Men	87.10 ± 23.78	88.16 ± 24.97	0.808	0.001		
**Maximal dynamic leg extension strength—1 RM (kg)**						
Women	52.14 ± 14.58	52.59 ± 9.81	0.747	0.001	0.435	0.004
Men	75.32 ± 15.49	71.36 ± 17.31	0.556	0.008		
**Maximum hand grip strength (left) (kg)**						
Women	21.06 ± 4.17	21.06 ± 3.76	0.923	0.000	0.051	0.027
Men	33.05 ± 7.40	37.33 ± 5.22	0.055	0.078		
**Maximum hand grip strength (right) (kg)**						
Women	22.39 ± 4.63	27.72 ± 4.12	0.564	0.004	0.197	0.012
Men	35.83 ± 7.20	38.11 ± 7.27	0.292	0.024		
**Maximum hand grip strength (the highest) (kg)**						
Women	22.84 ± 4.39	22.56 ± 3.86	0.856	0.000	0.090	0.021
Men	36.15 ± 7.03	39.56 ± 6.13	0.114	0.053		
**Arm curl test (rep)**						
Women	18.60 ± 3.94	18.33 ± 4.06	0.818	0.001	0.336	0.007
Men	16.45 ± 3.40	17.67 ± 1.73	0.213	0.034		
**Chair stand test (rep)**						
Women	17.26 ± 3.36	17.16 ± 3.10	0.993	0.000	0.153	0.015
Men	16.50 ± 3.68	18.44 ± 4.75	0.122	0.051		
**Step-in-place (steps)**						
Women	102.34 ± 16.86	103.03 ± 18.20	0.759	0.001	0.503	0.003
Men	107.83 ± 20.07	113.67 ± 18.83	0.398	0.016		
**8 foot up and go test (s)**						
Women	5.31 ± 1.10	5.33 ± 0.91	0.980	0.000	0.988	0.000
Men	5.18 ± 1.25	5.20 ± 10.5	0.903	0.000		

Data are presented as mean ± standard deviation; All analyzes are adjusted by age; *p*-value: differences between groups (yes vs. no) in each sex by one-way ANOVA; *p*-value (Gxsex): group-by-sex interaction (*p* < 0.05, all such occurrences); Two-factor repeated-measures ANOVA; 1 RM: one maximal repetition; rep: repetition; s: seconds.

**Table 4:** Strength and functional capacity between normal and obesity according fat percentage in men and women older than 70 years.

Sex	Fat Percentage		*p*-Value	η^2^ *p*	*p*-Value (Gxsex)	η^2^ *p*
	Normal	Obesity				
**Maximal isometric biceps strength (kg)**						
Women	21.26 ± 4.72	19.47 ± 5.72	0.174	0.020	0.358	0.006
Men	36.17 ± 9.04	32.63 ± 7.17	0.043	0.086		
**Maximal dynamic biceps strength—1 RM (kg)**						
Women	12.58 ± 4.18	14.20 ± 5.27	0.246	0.015	0.601	0.002
Men	33.32 ± 9.68	34.42 ± 8.91	0.718	0.003		
**Maximal isometric leg extension strength (kg)**						
Women	62.01 ± 17.08	56.62 ± 15.39	0.136	0.024	0.426	0.005
Men	85.98 ± 26.10	88.00 ± 22.79	0.841	0.001		
**Maximal dynamic leg extension strength—1 RM (kg)**						
Women	53.87 ± 13.83	51.89 ± 12.97	0.464	0.006	0.472	0.004
Men	72.43 ± 14.17	75.74 ± 15.73	0.994	0.000		
**Maximum hand grip strength (left) (kg)**						
Women	21.53 ± 2.63	20.94 ± 4.30	0.522	0.005	0.454	0.004
Men	32.94 ± 5.33	34.31 ± 8.05	0.941	0.000		
**Maximum hand grip strength (right) (kg)**						
Women	23.11 ± 3.96	21.92 ± 4.56	0.250	0.014	0.145	0.015
Men	34.71 ± 5.02	37.06 ± 8.06	0.542	0.008		
**Maximum hand grip strength (the highest) (kg)**						
Women	23.47 ± 3.51	22.56 ± 4.36	0.337	0.010	0.242	0.010
Men	35.53 ± 4.76	37.44 ± 7.85	0.668	0.004		
**Arm curl test (rep)**						
Women	18.84 ± 3.06	18.42 ± 4.17	0.630	0.003	0.948	0.000
Men	16.88 ± 2.89	16.56 ± 2.90	0.471	0.011		
**Chair stand test (rep)**						
Women	17.74 ± 2.23	17.09 ± 3.47	0.359	0.009	0.300	0.008
Men	17.94 ± 4.09	16.28 ± 3.76	0.039	0.089		
**Step-in-place (steps)**						
Women	107.95 ± 11.15	101.21 ± 18.27	0.100	0.029	0.871	0.000
Men	113.29 ± 18.97	106.56 ± 20.11	0.170	0.040		
**8 foot up and go test (s)**						
Women	4.97 ± 0.54	5.41 ± 1.12	0.085	0.032	0.513	0.003
Men	5.15 ± 1.67	5.21 ± 0.90	0.333	0.020		

Data are presented as mean ± standard deviation; all analyses are adjusted by age; *p*-value: differences between groups (low vs. normal) in each sex by one-way ANOVA; *p*-value (Gxsex): group-by-sex interaction (*p* < 0.05, all such occurrences): Two-factor repeated-measures ANOVA; 1 RM: one maximal repetition; rep: repetition; s: seconds.
